# Environmental Dynamics and Digital Transformation in Lower-Middle-Class Hospitals: Evidence from Indonesia

**DOI:** 10.3390/healthcare14020182

**Published:** 2026-01-12

**Authors:** Faisal Binsar, Mohammad Hamsal, Mohammad Ichsan, Sri Bramantoro Abdinagoro, Diena Dwidienawati

**Affiliations:** 1 Faculty of Economics and Business, Universitas Muhammadiyah Berau, Tanjung Redeb 77315, Indonesia; faisal_binsar@umberau.ac.id; 2 Management Department, Doctor of Research in Management, Binus Business School, Bina Nusantara University, Jakarta 11480, Indonesia; mhamsal@binus.edu (M.H.); sabdinagoro@binus.edu (S.B.A.); 3 Business Management Program, Binus Business School Undergraduate Program, Binus University, Jakarta 11480, Indonesia; 4 Management Program, Binus Business School Undergraduate Program, Binus University, Jakarta 11480, Indonesia; diena.t@binus.edu

**Keywords:** digital capability, environmental dynamism, digital transformation, technology adoption, healthcare technology

## Abstract

**Highlights:**

**What are the main findings?**

- Environmental dynamism shows only a weak positive relationship with digital capability among lower-middle-class hospitals in Indonesia, indicating that external pressure alone does not ensure successful digital transformation.
- Financial limitations, inadequate ICT infrastructure, and uneven staff training remain the primary barriers preventing hospitals from fully implementing electronic medical records and other digital systems.

**What are the implications of the main findings?**

- Policymakers should complement regulatory mandates with targeted financial incentives and capacity-building initiatives to strengthen hospitals’ digital readiness.
- Hospital managers must enhance internal leadership, budgeting priorities, and collaboration with technology partners to accelerate equitable digital transformation in the healthcare sector.

**Abstract:**

**Background/Objectives**: Digital transformation is increasingly essential for healthcare organizations to improve operational efficiency and service quality. However, in developing countries such as Indonesia, many lower-middle-class hospitals lag due to limited financial, human, and infrastructural resources. This study examines how environmental dynamism—comprising regulatory changes, market pressures, and technological shifts—affects the digital capabilities of these hospitals. **Methods**: A quantitative, cross-sectional survey was conducted in Class C and D hospitals across Indonesia. Respondents included hospital directors, deputy directors, and IT heads. Data were collected through structured questionnaires measuring environmental dynamism and digital capability using a six-point Likert scale. Reliability testing yielded Cronbach’s alpha values above 0.96 for both constructs. Correlation analysis was performed to examine the relationship between environmental dynamism and digital capability. **Results**: Findings reveal a weak positive correlation (*r* = 0.1816) between environmental dynamism and digital capability. Although external factors such as policy regulations and technological competition encourage digital adoption, hospitals with limited internal resources struggle to translate these pressures into sustainable transformation. Key challenges include low ICT budgets, inconsistent staff training, and insufficient infrastructure. **Conclusions**: The results suggest that environmental change alone cannot drive digital readiness without internal capacity development. To foster resilient digital healthcare ecosystems, policy interventions should integrate regulatory frameworks with practical support programs that strengthen resources, leadership, and human capital in lower-middle-class hospitals.

## 1. Introduction

The implementation of digital transformation in the healthcare industry has emerged as a vital concern globally [1], including in Indonesia [2]. Advances in information and communication technology (ICT) have influenced multiple aspects of hospital operations [3], ranging from medical data management to patient services. However, lower-middle-class hospitals in Indonesia continue to face significant challenges in adopting digital capabilities due to restricted resources and limited technological capacity [4,5]. Although digital technology has been shown to improve operational efficiency and healthcare quality [2,3], its adoption remains uneven across hospitals, particularly those operating under resource constraints. External influences such as regulatory demands, shifting patient expectations, and rapid technological change further shape hospitals’ digital transformation trajectories [6].

Understanding how lower-middle-class hospitals respond to these pressures is crucial, particularly in dynamic environments characterized by rapid change and uncertainty [6]. Environmental dynamism refers to unpredictable and swift alterations in the external environment, including changes in government regulation, technological development, and patient behavior [7]. These shifts introduce both challenges and opportunities, compelling hospitals to redefine digital strategies and organizational processes [8].

Although digital transformation in healthcare has been widely examined, prior research has predominantly focused on large or well-resourced hospitals, while empirical evidence from lower-middle-class hospitals remains limited [9]. Few studies have investigated how environmental dynamism interacts with internal digital capacity in healthcare institutions operating under significant resource constraints. Furthermore, existing research tends to emphasize technological adoption outcomes rather than the conditions shaping digital readiness [10]. This gap is particularly evident in developing countries, where environmental pressures and the development of digital capabilities occur simultaneously within complex operational contexts.

Grounded in digital capability theory and environmental contingency perspectives, this study addresses these gaps by examining hospitals in Indonesia that must navigate regulatory uncertainty, market shifts, and technological acceleration [6,7]. Specifically, we investigate the extent of digital capacity adoption in lower-middle-class hospitals, the relationship between environmental dynamism and capability development, and the significant barriers to digital transformation in this setting. Drawing on these perspectives, the study addresses the following research questions: (a) What is the extent of digital capacity adoption in lower-middle-class hospitals in Indonesia? (b) Can the level of environmental dynamism impact hospitals’ ability to adapt to digital technology? (c) What are the primary obstacles to the use of digital technology in this group of hospitals? Together, these questions explore how external dynamics shape digital preparedness and organizational adaptation in resource-limited hospitals [11,12,13].

This study employs a quantitative survey approach across hospitals in Indonesia, using descriptive and correlational analyses to examine patterns of digital capacity and the extent to which environmental dynamism relates to capability development. Additional analysis identifies key barriers hindering adoption, offering insight into how contextual conditions shape digital readiness in resource-constrained settings. Findings contribute to the literature by providing empirical evidence from underrepresented healthcare institutions and illustrating how environmental factors interact with organizational capability. From a practical perspective, the study offers implications for policymakers and hospital administrators seeking to support inclusive digital transformation.

The remainder of this article is structured as follows: Section 2 reviews the literature on digital capability and environmental dynamism; Section 3 outlines the survey design and analytical procedures; Section 4 present quantitative findings and their interpretation; and Section 5 summarizes key insights and implications.

## 2. Literature Review

Resource-Based Theory (RBT) [14,15], Environmental Contingency Theory [16], and Diffusion of Innovation Theory [17] form the conceptual foundations of this study. RBT emphasizes that an organization’s competitive advantage emerges from internal capabilities such as digital competency, encompassing technological infrastructure and human capital [14]. Environmental Contingency Theory argues that organizations must adjust their internal capabilities to respond effectively to external pressures, including regulatory change and shifting market demands [18,19]. Diffusion of Innovation Theory further explains how new technologies are adopted across organizations, identifying factors that support or hinder digital integration [20]. Together, these frameworks support the examination of how hospitals in resource-constrained environments navigate digital transformation under dynamic external conditions.

Digital capability relates to an organization’s preparedness to adopt and integrate digital systems, supported by technological resources, competent human capital, and managerial commitment [21,22]. Prior evidence shows that digital systems such as HIS, telemedicine, and EMR can improve service quality, operational efficiency, and data administration [21]. However, lower-middle-class hospitals face greater challenges in establishing these capabilities due to resource limitations, infrastructure gaps, and operational constraints [23].

Environmental dynamism refers to uncertainty, speed, and complexity in external ecological change, which may stem from technological shifts, regulatory reforms, and market dynamics [24]. In the hospital context, rapidly changing digital policies, technology platforms, and patient service expectations can either accelerate or obstruct digital transformation [25]. Some studies suggest that dynamic environments stimulate digital capability development [26], whereas organizations lacking resources may struggle to respond.

In Indonesia, lower-middle-class hospitals are susceptible to regulatory shifts relating to HIS implementation, EMR adoption, and digital reporting systems [27,28,29]. Financial constraints further limit hospitals’ ability to build and maintain compatible systems [30]. Hospitals also face competitive benchmarking pressure, as technologically advanced institutions shape expectations for telemedicine, scheduling, and monitoring systems [31,32]. These comparative pressures highlight disparities in digital maturity that stem from resource levels rather than strategic preferences [27].

Environmental dynamism is also shaped by shifting patient expectations for faster, digital access to health information [33]. National policies such as the JKN program require digital documentation and claims management, creating additional layers of operational and administrative complexity [33,34]. Thus, environmental demands intersect with capability constraints, intensifying transformation challenges in hospitals with limited capacity.

Prior studies confirm that digital technology adoption improves hospital performance, especially in operational efficiency and service quality [33,35]. However, adoption rates differ significantly across organizational types, with hospitals in resource-limited settings progressing more slowly [36]. This disparity raises questions about how lower-middle-class hospitals adapt to regulatory and technological change, and whether external dynamism serves as a catalyst or impediment to digital capability development [8].

The literature also identifies significant barriers to digital implementation, including financial constraints, change resistance, and inadequate policy support [37]. Government interventions do not consistently reduce capability gaps, particularly for hospitals with limited infrastructure [38,39]. These findings suggest misalignment between environmental pressures and organizational capacity.

Despite extensive work on digital healthcare transformation, few empirical studies have examined how environmental dynamism relates to internal digital capability in resource-constrained hospitals, particularly in developing countries. Existing research emphasizes transformation outcomes but rarely examines how external dynamics shape the formation of readiness and capability. Therefore, this study investigates whether environmental dynamism is associated with levels of digital capability among lower-middle-class hospitals in Indonesia.

Drawing on Environmental Contingency Theory and digital capability perspectives, we propose the following hypothesis:

**H1.**  *Environmental dynamism is positively associated with digital capability in lower-middle-class hospitals in Indonesia*.

This hypothesis aligns theoretical expectations with the study’s research objective and provides a measurable basis for empirical testing.

## 3. Methods

### 3.1. Study Design

This study employed a descriptive–correlational research design [40] to examine the association between digital capability and environmental dynamism among lower-middle-class hospitals (Class C and D) in Indonesia. A total of 285 hospitals participated in the research, representing facilities across multiple provinces. Data were collected via a cross-sectional online survey administered via Google Forms throughout 2023.

The survey instrument consisted of two sections. The first captured respondent demographics and hospital characteristics; the second contained measurement items for the two primary variables: Digital Capability and Environmental Dynamism. A six-point Likert scale, ranging from 1 (Strongly Disagree) to 6 (Strongly Agree), was used to measure responses consistently across all items [41]. The six-point scale was intentionally selected to reduce neutral or midpoint bias and to encourage clearer attitudinal responses.

Measurement items for digital capability were adapted from existing literature [21,22], covering technological infrastructure readiness, human resource digital competency, and management support. To ensure internal construct coherence, items were structured around established conceptual dimensions rather than using mixed indicators. Environmental dynamism indicators were operationalized from validated theoretical constructs in prior research [24,26], specifically reflecting uncertainty, regulatory change, and technological advancement. Only items conceptually linked to dynamism were included to avoid construct ambiguity or dimension drift.

Overall, this research design provides a holistic depiction of digital readiness and external dynamic pressures among lower-middle-class hospitals in Indonesia. Table 1 presents the detailed measurement items for each construct.

As with most survey-based studies, data reflect self-reported perceptions rather than objective performance indicators. This introduces the possibility of response bias, including social desirability, selective recall, and overestimation of organizational capability. To mitigate this risk, items were designed using neutral language, and respondents were assured anonymity; however, some degree of subjective bias remains possible.

This study adhered to ethical guidelines for human-subject research. According to institutional and national regulations, ethical board approval was not required for minimal-risk anonymous survey research. Participation was voluntary, informed consent was obtained digitally before survey submission, and no identifiable personal data were collected.

### 3.2. Participants

Participants included senior hospital leaders, such as directors, vice directors, and heads of digital systems or information technology units. These individuals were selected because their roles involve direct responsibility for strategic decisions related to digital technology adoption and institutional development [46].

A purposive sampling technique was used to select hospitals officially registered as Class C or D within the Indonesian Ministry of Health system. Inclusion criteria required participants to (1) hold a senior decision-making position, (2) possess relevant knowledge of hospital digital operations, and (3) be actively involved in policy or implementation processes related to digital transformation. This approach ensured that participants could provide informed, organization-level perspectives that reflect institutional digital capability and environmental influences.

Because participation relied on voluntary response from senior leaders, the data may disproportionately reflect hospitals with higher managerial engagement in digital transformation, potentially limiting representativeness.

### 3.3. Statistical Analysis

Survey data were collected and organized in Microsoft Excel before analysis in Python (version 3.10). Multiple Python libraries were used, including pandas for data processing, numpy for matrix operations, scipy for correlation testing, and matplotlib for visualization [47].

Data cleansing procedures included removing incomplete responses through listwise deletion, validating variable entry formats, and flagging outliers exceeding ±3 standard deviations. Outlier decisions were made based on distribution patterns and response consistency to preserve the validity of the data structure.

Descriptive statistics (frequency, percentage, mean, standard deviation) were used to characterize respondent and hospital profiles. This study then conducted Pearson correlation analysis to assess the association between digital capability and environmental dynamism. Because the objective of this research was not causal inference, but somewhat exploratory association mapping, correlation was selected as the most appropriate analytical strategy [40]. Regression or causal modeling was not conducted.

The strength and significance of correlations were evaluated using *r*-values, *p*-values, and standard effect size interpretation. No control variables were included, as the design aimed to explore relationships among variables rather than to construct predictive or causal models.

Internal consistency for both constructs was tested using Cronbach’s alpha. Although construct validity tests, such as CFA or AVE, were not conducted, reliability results indicated strong internal coherence, and the measurement items were theoretically grounded. This approach was consistent with the descriptive–correlational scope of the research.

The analytical strategy was correlational and did not permit causal inference. The absence of regression or multivariate control limits analytical depth, as potential confounding variables (e.g., hospital size, ownership type, funding model, or IT expenditure) were not statistically isolated. This approach prioritizes exploratory insight at the expense of explanatory precision.

## 4. Results and Discussion

### 4.1. Respondent

This study included a sample of 285 high-ranking executives from lower-middle-class hospitals in Indonesia. The sample comprised individuals at different hierarchical levels and with varying lengths of service at the hospital. According to the respondent profile data presented in Table 2, the most significant proportion of respondents are individuals in Director or Head of Hospital positions, accounting for 78.9% of the total. Among them, 25.3% have served for less than 1 year, 47.4% have served between 1 and 5 years, and 6.3% have served for more than 5 years. The significant presence of Hospital Directors/Heads indicates that the adoption of digital technology in hospitals is heavily affected by those with the most important level of authority in hospital operations [48].

Furthermore, 7.7% of participants held the position of Deputy Director, and among them, 6.3% had served for 1 to 5 years. This demonstrates that the Deputy Director, although to a lesser extent than the Director, also plays a substantial role in strategic decisions regarding information and communication technology (ICT) in hospitals. Concurrently, the individual in charge of the IT Division, which comprises 13.3% of the workforce, bears significant responsibility, particularly given their technical expertise and direct involvement in implementing digital technologies. Based on the data, the majority of Heads of IT Divisions have a tenure of 1–5 years (7%), while an additional 2.8% have served for more than 5 years. This indicates a considerable level of expertise in overseeing hospital IT infrastructure.

Regarding hospital classification, Table 3 shows that 45.26% of participants were from class C hospitals, whereas 54.74% were from class D hospitals. These findings indicate that the majority of hospitals included in this study are classified as lower socioeconomic class (class D), which often have fewer resources than higher-class hospitals. This profile is highly pertinent to research on digital capabilities and environmental dynamics in lower-middle-class hospitals. These hospitals, which have limited resources, are expected to undergo digital transformation to enhance the efficiency and quality of their services.

This respondent profile indicates a significant presence of influential individuals responsible for key decisions in hospitals serving the lower-middle class. The bulk of these individuals possess ample expertise in their respective roles. Understanding how strategic ideas and policies on digital technology adoption are influenced by individuals’ knowledge and position within the hospital hierarchy is crucial [49].

According to Table 4, the hospitals participating in this study have generally been in operation for a substantial period. Approximately 56.5% of hospitals have been in operation for over a decade. This suggests that most of these hospitals have successfully adapted to changes in the regulatory environment and service requirements, including the National Health Insurance (JKN) policy and their collaboration with BPJS Health. Hospitals with a history of over ten years typically possess greater expertise in navigating external factors, such as shifts in government legislation concerning Hospital Management Information Systems (HIS) and service standardization.

When comparing hospital classes, it is evident that class C hospitals are often more established, with 32.6% having been in operation for more than 10 years. In contrast, just 23.9% of class D hospitals have an operational history of more than 10 years. On the other hand, class D hospitals have a higher proportion of patients in the younger age group, specifically ages 5–10 (24.2%). This demonstrates that class D hospitals, despite being relatively new, are still developing their capacity to address operational challenges and comply with regulatory requirements.

According to the data, most hospitals in this study have undergone accreditation. Specifically, 37.5% of hospitals have achieved the primary level, while 15.8% have reached the highest level, known as the plenary level, in the Indonesian hospital accreditation system. Among hospitals in the high-accreditation category, the majority (20%) are classified as Class C; 57 hospitals achieved primary-level accreditation, and 33 achieved plenary-level accreditation. On the other hand, class D hospitals often have a lesser status, with 18.6% operating at an intermediate level and only 4.2% achieving a plenary level.

The presence of 8 class D hospitals that remain unaccredited suggests that smaller or resource-constrained hospitals face significant difficulties in achieving quality and patient safety standards. This demonstrates a disparity in the quality of service and infrastructure preparedness between Class C and Class D hospitals.

Overall, these data demonstrate that most hospitals in our study are committed to improving the quality of healthcare services through certification. Accreditation serves as evidence that a hospital has adhered to the government’s established criteria for medical services, patient safety, and hospital administration. Hospitals’ efforts to attain top-tier accreditation at both primary and plenary levels demonstrate a resolute commitment to staying abreast of technological advancements and legal developments, particularly in the context of digital transformation in the healthcare sector.

The hospitals involved in this study illustrate the preparedness and challenges faced by lower-middle-class hospitals in Indonesia in implementing digital technology and meeting more stringent certification criteria. Established hospitals typically possess superior operational capabilities and have made substantial investments in enhancing quality and technology [12]. In contrast, younger or non-accredited hospitals face greater challenges in their efforts to undertake digital transformation.

### 4.2. The Correlation Between Digital Capability and Environmental Dynamism

Table 5 displays the range of values for the Digital Capability question, with an average (mean) value ranging from 3.05 to 4.65. This suggests that the majority of respondents perceive a moderate-to-high level of digital capability within their hospital. This diagram illustrates the adoption of digital technology in hospitals, with varying degrees of deployment. There is a specific location, referred to as DC6, where the average value is comparatively lower. This item concerns the insufficient variables supporting budget allocation pledges for ICT in several hospitals. The standard deviation ranged from 1.09 to 1.46, suggesting differences in perceptions among participants. The discrepancy in question may stem from factors such as hospital size, IT team capabilities, or the rate at which each hospital adopts technology. The digital capacity of lower-middle-class hospitals in Indonesia shows promise, albeit with notable variation across facilities. This competence includes the utilization of digital applications, the number of computers, and the number of IT staff. These factors are crucial metrics for evaluating a hospital’s preparedness to embrace digital transformation [50].

The mean ED item score ranges from 3.38 to 4.65, indicating that hospital executives attach significant importance to the external environment, including regulatory changes, market demands, and technological advancements, when making strategic decisions. However, certain hospitals perceive a greater degree of influence from the external environment than others. ED4 and ED5 have much higher average values (4.59 and 4.65), suggesting that external influences, such as the use of the vClaim application (BPJS), which is mandatory in hospitals, have a substantial impact on hospitals. The range of standard deviation, which falls between 1.29 and 1.59, indicates a significant variation in the perceptions of environmental dynamics among respondents. Variables such as the hospital’s geographical location (urban or rural) or accreditation status may influence this variation. Indonesian hospitals face a constantly changing external environment characterized by legislative changes, technological advancements, and economic conditions. The diversity of opinions among participants indicates that some hospitals face more complex environmental barriers, which can affect their approach to and acceptance of digital technologies.

The reliability analysis confirmed strong internal consistency for both constructs, with Cronbach’s alpha values of 0.96032420 for Digital Capability and 0.962472157 for Environmental Dynamism. These results indicate that the survey items exhibit acceptable reliability for further statistical analysis.

A Pearson correlation test was then conducted to examine the relationship between Digital Capability and Environmental Dynamism. The results are presented in Table 6.

The analysis indicates a weak but statistically significant positive relationship between Environmental Dynamism and Digital Capability (*r* = 0.181586, *p* = 0.002), meaning that hospitals experiencing higher levels of external change tend to report slightly stronger digital capability. However, the small effect size indicates that environmental conditions alone account for only a limited proportion of the variance in digital readiness. This suggests that internal organizational factors—such as available budget, IT workforce expertise, infrastructure maturity, and leadership commitment—may play a more substantial role than environmental pressures in shaping capability development.

These findings align with research conducted in other resource-constrained healthcare settings. For instance, similarly weak relationships were reported when examining digital adaptation in public hospitals under regulatory pressure, suggesting that external demands do not necessarily drive internal capability change [51,52]. In contrast, recent work on digital orientation and technological turbulence shows that external environmental pressure strengthens digital outcomes primarily in organizations with strong internal resource bases. For example, a large-scale longitudinal study of S&P 500 firms demonstrated that technological turbulence amplified the positive effects of digital capability only among well-resourced organizations operating in complex competitive environments [53]. Together, these comparisons indicate that environmental stimuli may contribute to digital progress only when organizational readiness and resource capacity are sufficiently developed.

In the context of Indonesian lower-middle-class hospitals, the weak correlation underscores that external regulatory and technological pressures have not yet translated into substantial improvements in digital capability. Persistent structural barriers, such as financial constraints, shortages of qualified IT personnel, limited infrastructure, and uneven EMR implementation [6,7], continue to impede adoption, particularly in rural settings. These results reinforce the view that successful digital transformation depends not only on environmental pressure but also on institutional resource strength and strategic investment, highlighting the need for tailored policy support and capacity-building initiatives.

### 4.3. Digital Capabilities

**Figure 1:**
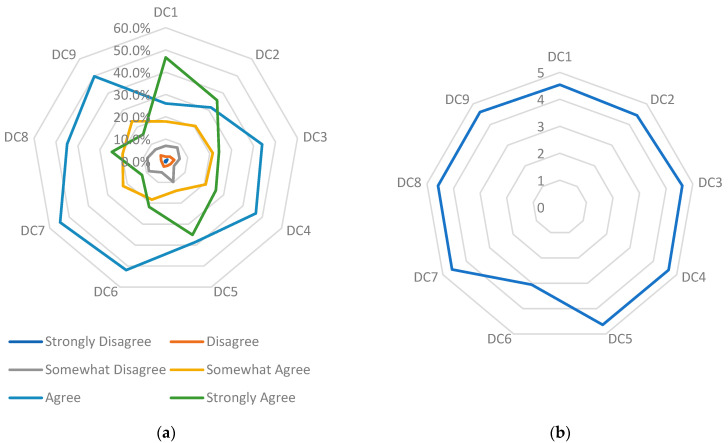
Respondents’ answers to each Digital Capability item (**a**) percentage per choice, (**b**) average choice. Source(s): Authors’ work.

Lower-middle-class hospitals in Indonesia have made meaningful progress in implementing digital capabilities, particularly in digital workflow, reporting processes, and workload reduction. These improvements appear to enhance operational efficiency, streamline administrative activities, and support faster and more accurate reporting. Figure 1 shows high agreement across most items, indicating broad acceptance of digital tools to support daily hospital operations and service delivery.

Despite these advances, ICT funding remains a significant constraint. Item DC6 shows wide variability in responses, indicating uneven financial capacity to support digital infrastructure. Limited funding continues to hinder the sustainability and expansion of digital capabilities, underscoring the need for stronger internal budgeting strategies and external financial support mechanisms.

As illustrated in Figure 1a, the distribution of responses shows an intense concentration in the ‘Agree’ and ‘Strongly Agree’ categories across most items, particularly DC1, DC3, DC4, DC5, and DC7. This pattern visually reinforces the numerical findings in Table 5, indicating high perceived digital capability and alignment between leadership support, workflow optimization, and operational digitalization. In contrast, DC6 stands out with the lowest proportion of positive responses and the widest spread across disagreement categories. This visual gap shows clear divergence in hospital readiness for ICT budget allocation, suggesting uneven investment capacity across facilities. Figure 1b further supports this pattern, with DC6 forming a sharp inward deviation relative to other indicators, visually confirming its status as a capability bottleneck. This combination of visual and statistical evidence highlights structural constraints in resource allocation despite strong operational digitalization momentum, a pattern consistent with findings in resource-limited hospital settings reported by Lu et al. [54] and Andrade et al. [55].

Furthermore, hospital leadership has demonstrated significant support for the digital transformation project [48], as evidenced by the outcomes for item DC4. This support is crucial for motivating people to use technology to its full potential. Furthermore, the presence of certification among employees in the digital technology industry, as evidenced by the DC3 findings, indicates the implementation of initiatives to enhance workforce skills and capabilities. This accreditation is crucial to guarantee that staff possess sufficient proficiency in utilizing digital systems. However, the results also indicate potential for improvement, particularly in broadening the range of information and communication technology (ICT) training available to employees. This is particularly evident in DC2, where some respondents still perceive the training provided to employees as ineffective. This implies that despite the presence of training programs, there is a need to enhance the quality and pertinence of training to ensure fairness and effectiveness in equipping employees with essential digital skills.

Overall, the majority of respondents expressed consensus that digital technology enhances hospital operational efficiency. In addition to alleviating hospital employees’ workload, technology contributes to cost savings and streamlines reporting processes, such as the implementation of Hospital Information System (SIRS) reporting. Nevertheless, to ensure the continued advancement of hospital digital capabilities, two specific issues must be prioritized. Initially, it is essential to increase financial allocations to bolster the technological infrastructure. Lack of adequate financial backing can impede the growth of digital capabilities [27]. Furthermore, it is imperative to ensure that ICT training is distributed equitably across all hierarchical levels, thereby equipping each individual with the competencies necessary to leverage technology fully. By following these procedures, lower-middle-class hospitals in Indonesia will enhance their readiness to meet the demands of the digital era and deliver more effective, high-quality healthcare services.

### 4.4. Environmental Dynamism

**Figure 2:**
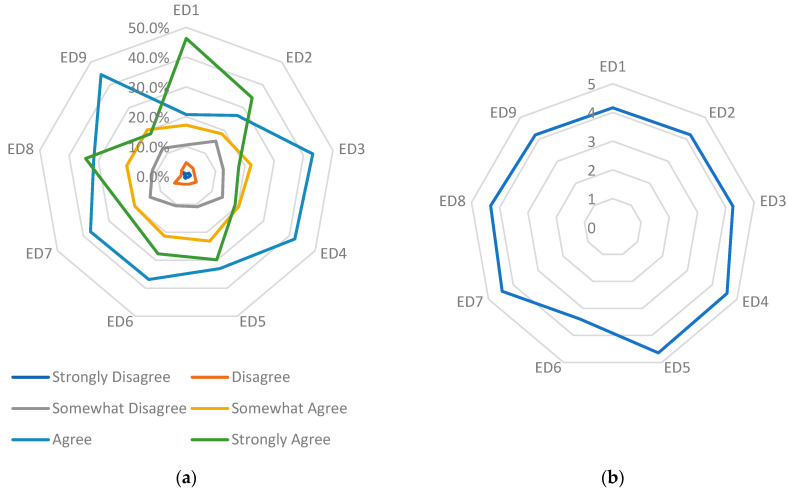
Respondents’ answers to each Environmental Dynamism item (**a**) percentage per choice, (**b**) average choice. Source(s): Authors’ work.

Figure 2 illustrates a wide variation in perceptions of environmental dynamism across lower-middle-class hospitals in Indonesia. Items ED4, ED5, and ED7 show the highest levels of agreement, indicating that respondents perceive regulatory changes—particularly those related to BPJS vClaim platforms and EMR requirements—as major external drivers of operational change. This pattern aligns with national digital health policy initiatives mandating EMR adoption, as outlined in PMK No. 24 of 2022 [29].

In contrast, ED6 shows lower mean scores and a wider spread of responses, suggesting uneven readiness to adopt biometric identification technology. This is consistent with evidence that hospitals with limited digital infrastructure face constraints in implementing advanced identification systems [56,57]. The contrast between high agreement on regulatory items (ED4, ED5, ED7) and low agreement on technological readiness (ED6) indicates that external pressure alone may not ensure capability development—particularly where financial, technical, and human resource limitations persist. These findings support the broader interpretation that, while environmental expectations are rising, hospitals’ internal readiness varies substantially, particularly in low-resource settings.

Regarding the ED1 item (Patients seeking internet information), a substantial majority of respondents indicated strong agreement: 46.3% strongly agreed, and 20.7% agreed. This demonstrates hospitals’ recognition of the importance of providing online information to patients, including details on physicians’ practice schedules and room availability. This tendency indicates that individuals are increasingly demanding prompt and effective access to information through digital technologies. With an average of 4.16, the majority of hospitals recognize the importance of meeting this demand to remain relevant in delivering treatments that accommodate the digital requirements of contemporary patients.

Nevertheless, we encountered difficulties in ED6 (where our hospital is equipped to use fingerprints for patient identification), which resulted in lower consensus, averaging only 3.38. Only 27.7% of participants expressed strong agreement with the preparedness of their hospital to implement fingerprint identification technology. In contrast, 21.4% partially agreed, and 10.5% partially disagreed. This indicates that the digital infrastructure in many hospitals is not yet optimal, particularly with respect to patient identification technologies. Hospitals in the lower-middle class may encounter technical or budgetary barriers when attempting to implement more sophisticated technologies, which can have an impact on the effectiveness and precision of patient data management [58].

Within the context of ED7 (government legislation, namely PMK No. 24 of 2022 regarding Medical Records [29], which mandates the use of digital technology in hospitals), the level of approval is significantly elevated, with an average rating of 4.45. This indicates that a significant number of hospitals recognize that government regulations promote the adoption of digital technology, and approximately 37.2% concur that these restrictions directly affect their hospital operations. The implementation of such rules undoubtedly motivates hospitals to undertake digital transformation; however, the degree of preparedness for this change may vary across hospitals.

However, ED3 (the impact of digital technology support in other hospitals on the frequency of patient visits to our hospital) also yields intriguing findings, with an average of 4.24. Approximately 43.2% of participants agreed that the level of rivalry among hospitals in adopting digital technologies affected the volume of patient visits. This indicates that hospitals are beginning to see the impact of technology-driven competition [59]. In the healthcare sector, the integration of advanced technologies can play a crucial role in patient recruitment.

Responses to ED4, which pertain to the requirement of running the BPJS vClaim Application in our hospital, exhibited a significantly high degree of agreement with a mean score of 4.59. A significant proportion of respondents, specifically 42.1%, agreed, while 18.9% strongly agreed, that the implementation of this application is necessary. This suggests that the adoption of digital transformation within the BPJS system has become a necessity in numerous hospitals. This underscores the necessity of a robust digital infrastructure capable of meeting the operational requirements of a digitally managed national healthcare system.

In summary, these findings indicate that the external environment, particularly with respect to technology and regulation, necessitates greater responsiveness among lower-middle-class hospitals to digital transformation. Nevertheless, the primary obstacle encountered is the preparedness of hospitals to embrace more sophisticated technologies and adjust their operations to comply with new regulatory requirements [60]. Government involvement and collaboration with other entities can significantly help ensure that these hospitals meet expectations regarding both technological advancements and patient care.

## 5. Conclusions

While some lower-middle-class hospitals in Indonesia have made notable strides in adopting digital health technologies, many remain in the early stages of transformation due to persistent financial and infrastructure constraints. Although environmental dynamism—such as shifting regulations and increasing digital expectations—plays a significant role, our findings suggest that such pressures alone do not ensure technological readiness. The weak but significant association identified in this study indicates that external change does not automatically translate into internal capability growth, particularly in resource-constrained settings. This highlights a critical gap in the adoption of frontier technologies in hospitals with limited organizational capacity.

This research contributes to the literature by empirically linking environmental dynamism and digital capability in under-resourced hospitals, providing evidence from a developing-country context that remains underrepresented in digital health studies. Unlike findings from larger, well-funded healthcare systems, our results indicate that internal readiness factors more strongly shape capability development than external pressure alone. These insights expand the theoretical understanding of drivers of digital transformation in emerging economies and reinforce the need for capacity-based digital health strategies.

In practice, the findings inform policymakers of the importance of coupling regulatory mandates—such as electronic medical record implementation—with concrete support mechanisms, including financial incentives, infrastructure grants, and ICT training programs. For hospital leaders, the study underscores the importance of internal investment in IT workforce competencies, infrastructure maturity, and leadership engagement. By aligning environmental pressure with organizational readiness, lower-middle-class hospitals may accelerate digital adoption and strengthen system resilience in the face of future disruptions.

In relation to the research objective, this study demonstrates that while external regulatory and technological pressures are associated with digital capability, internal resource capacity remains the primary determinant of digital readiness in lower-middle-class hospitals. These findings provide empirical clarity to a previously underexplored question: whether environmental dynamism alone can drive digital transformation in under-resourced healthcare settings. Methodologically, the cross-sectional, self-reported survey design may have limited the ability to infer causality or to capture organizational change over time, suggesting that future studies could employ longitudinal or mixed-methods approaches to gain deeper insight into capability formation dynamics.

## 6. Limitations and Further Research

It is essential to recognize that this study has several constraints. This research is cross-sectional, as the data were obtained at a single point in time. This approach limits the ability to observe changes or progress in digital capabilities and environmental dynamics over time. Longitudinal studies are more effective in understanding the long-term development of external environmental changes and the use of digital technologies. Moreover, this study examines hospitals in the lower-middle class (classes C and D) in Indonesia. Therefore, the findings of this study may not be entirely applicable to hospitals in the upper-class category (classes A and B) or to healthcare systems in other countries with distinct health dynamics. Disparities in resources, legislation, and economic situations can affect the applicability of these conclusions.

In addition to these limitations, the analytical approach used in this study relied on bivariate correlation without incorporating control variables or examining causal direction. This restricts the ability to rule out potential confounding factors and limits the depth of inference that can be drawn from the statistical results. Furthermore, the use of self-reported survey data may introduce perceptual and social desirability biases, as well as overestimation of digital capability—particularly given that respondents held senior strategic roles. These factors may influence response patterns and should be considered when interpreting the findings.

To further investigate, future research may examine the long-term effects of digital capability and environmental dynamism on overall hospital performance, including financial outcomes and patient satisfaction. Additional variables, such as government support, private-sector involvement, and technology readiness, could be examined to deepen understanding of the challenges of digital transformation in the healthcare sector. A mixed-methods approach that integrates quantitative analysis with interviews or case studies may also provide deeper insights into barriers and solutions. Future studies may employ multivariate statistical models or regression-based approaches to assess predictive relationships and more rigorously evaluate the contributions of internal and external factors. Introducing control variables, such as hospital size, ownership type, accreditation status, or funding level, may strengthen analytical precision. Longitudinal or panel-based survey designs could further clarify how digital capability evolves relative to environmental shifts, while integrating objective hospital performance metrics may reduce reliance on self-reported perceptions.

## Figures and Tables

**Table 1:** Measurement Instruments.

Variables	Indicators		Source
Digital Capabilities	DC1	We have sufficient ICT manpower	[42,43]
	DC2	Our employees are given education and training to be skilled in ICT.	
	DC3	Some of our employees already have digital certification in the health services field.	
	DC4	Our leaders encourage employees to utilize digital technology.	
	DC5	Our hospital has a digital service flow (such as registration, examination by a doctor, or taking medicine)	
	DC6	Our hospital has the support of a commitment to allocate sufficient budget for ICT.	
	DC7	The use of ICT in our hospitals reduces the workload of employees.	
	DC8	The use of ICT in our hospital saves operational costs.	
	DC9	ICT in our hospital makes it easier to make reports (such as SIRS reports).	
Environmental Dynamism	ED1	Patients want to know information online (such as doctors’ practice schedules or room availability)	[22,31,33,44,45]
	ED2	Intense developments, such as pandemics or endemics, limit the accumulation of patients in hospitals	
	ED3	Digital technology support at other hospitals influences the number of patient visits to our hospital.	
	ED4	The claim (BPJS) application must be run at our hospital.	
	ED5	The use of GPS in ambulances helps our hospital staff prepare for patient arrival.	
	ED6	Our hospital is ready to run fingerprints for patient identification.	
	ED7	Government regulations require our hospitals to use digital technology.	
	ED8	Drug taxes burden our patient care costs.	
	ED9	Complete pricing data is required at our hospital to support claim submission.	

**Table 2:** Respondents by Position and Length of Service.

Respondent’s Position	Length of Service			
	<1 Year	1 to 5 Years	>5 Years	Total Respondents
Director/Head of Hospital	72 (25.3%)	135 (47.4%)	18 (6.3%)	225 (78.9%)
Deputy Director	3 (1.1%)	18 (6.3%)	1 (0.4%)	22 (7.7%)
Head of IT Division	10 (3.5%)	20 (7.0%)	8 (2.8%)	38 (13.3%)
Total	85 (29.8%)	173 (60.7%)	27 (9.5%)	285 (100%)

**Table 3:** Number of respondents by hospital class.

RS Class	Number of Respondents	(%)
Class C	129	45.26%
Class D	156	54.74%

**Table 4:** Length of Hospital Operation and Hospital Accreditation by Class.

	RS Class					
	Class C		Class D		Total	
How long the hospital has been operating						
<5 Years	4	1.4%	19	6.7%	23	8.1%
5–10 Years	32	11.2%	69	24.2%	101	35.4%
>10 Years	93	32.6%	68	23.9%	161	56.5%
Hospital Accreditation						
Not yet accredited	0	0.0%	8	2.8%	8	2.8%
First Pass	10	3.5%	24	8.4%	34	11.9%
Basic Level	5	1.8%	9	3.2%	14	5.0%
Intermediate Level	24	8.4%	53	18.6%	77	27.0%
Main Level	57	20.0%	50	17.5%	107	37.5%
Plenary Level	33	11.6%	12	4.2%	45	15.8%

**Table 5:** Descriptive and Reliability Statistics.

Variables		Mean	Median	Standard Deviation	Cronbach’s Alpha
Digital Capabilities	DC1	4.54386	5	1.157799	0.960324206
	DC2	4.445614	5	1.014693	
	DC3	4.603509	5	1.187073	
	DC4	4.652632	5	1.169764	
	DC5	4.638596	5	1.168506	
	DC6	3.05614	2	1.464488	
	DC7	4.614035	5	1.09664	
	DC8	4.582456	5	1.176819	
	DC9	4.603509	5	1.141713	
Environmental Dynamism	ED1	4.164912	4	1.435527	0.962472157
	ED2	4.203509	5	1.361178	
	ED3	4.249123	4	1.587043	
	ED4	4.596491	5	1.377123	
	ED5	4.649123	5	1.38008	
	ED6	3.389474	3	1.403929	
	ED7	4.449123	5	1.454125	
	ED8	4.322807	5	1.292314	
	ED9	4.2	5	1.453020	

**Table 6:** Pearson Correlation Analysis: Digital Capability and Environmental Dynamism.

Variables	*r*-Value	*p*-Value	Effect Size	n
Digital Capabilities vs. Environmental Dynamism	0.181586	0.002	Small	285

## Data Availability

The data supporting the findings of this study are available in this link https://doi.org/10.5281/zenodo.18057580. Due to institutional privacy agreements and confidentiality considerations with participating hospitals, the dataset is not publicly available. Aggregated data and analysis outputs can be shared upon request for academic purposes.

## Abbreviations

The following abbreviations are used in this manuscript:

DC	Digital Capabilities
ED	Environmental Dynamism
EMR	Electronic Medical Records
HIS	Hospital Information Systems
ICT	Information and Communication Technology
RBT	Resource-Based Theory
